# Lavender-like cobalt hydroxide nanoflakes deposited on nickel nanowire arrays for high-performance supercapacitors†

**DOI:** 10.1039/c8ra02844c

**Published:** 2018-05-11

**Authors:** Jie Liao, Xuanyu Wang, Yang Wang, Songyang Su, Adeela Nairan, Feiyu Kang, Cheng Yang

**Affiliations:** Division of Energy and Environment, Graduate School at Shenzhen, Tsinghua University Shenzhen 518055 China yang.cheng@sz.tsinghua.edu.cn; School of Materials Science and Engineering, Tsinghua University Beijing 100084 China

## Abstract

Hierarchical nanostructured electrodes with excellent electronic properties and high specific surface areas have promising applications in high-performance supercapacitors. However, high active mass loading and uniform structure are still crucial in fabricating such architectures. Herein, Co(OH)_2_ nanoflakes were homogeneously deposited on nickel nanowire arrays (NNA) through a hydrothermal approach to form an NNA@Co(OH)_2_ (NNACOH) composite electrode. The as-synthesized one dimensional (1D) system had a lavender-like structure with a high mass loading of 5.42 mg cm^−2^ and a high specific surface area of 74.5 m^2^ g^−1^. Due to the unique electrode structure characteristics, the electrode could deliver a high specific capacitance of 891.2 F g^−1^ at the current density of 1 A g^−1^ (corresponding to an areal capacitance of 4.83 F cm^−2^ at 5.42 mA cm^−2^). The capacitance could still maintain a high value of 721 F g^−1^ when the current density is increased to 50 A g^−1^. In addition, the electrode showed superior cycle stability with a capacitance retention of 89.3% after charging/discharging at the current density of 10 A g^−1^ for 20 000 cycles. A flexible asymmetric supercapacitor (ASC) was assembled by employing NNACOH as the positive electrode and activated carbon (AC) as the negative electrode. It delivered a maximum energy density of 23.1 W h kg^−1^ at the power density of 712 W kg^−1^ and an energy density of 13.5 W h kg^−1^ at the maximum power density of 14.7 kW kg^−1^ (based on the total mass of the electrodes), showing the state-of-the-art energy storage ability of the Co(OH)_2_ cathode material at device level.

## Abstract 2

Co(OH)_2_ nanoflakes deposited on nickel nanowire arrays with a lavender-like architecture are prepared. Using it as cathode and activated carbon as anode, the flexible supercapacitor shows superior energy storage ability.

## Graphical abstract

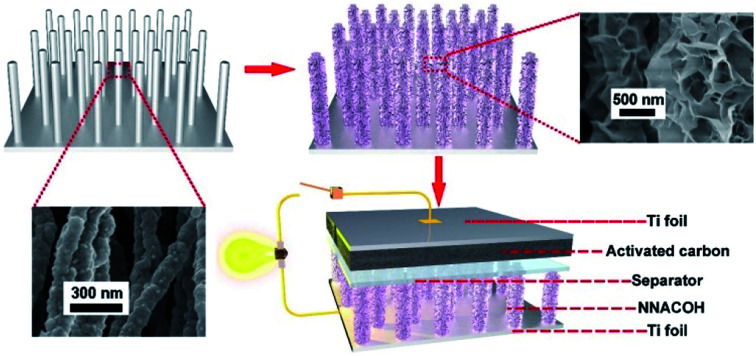

## 1 Introduction

To address the critical challenges of both the energy crisis and deteriorating ecological environment, great efforts have been devoted to developing clean and sustainable energy storage technologies such as batteries and electrochemical capacitors. Electrochemical capacitors, also known as supercapacitors, have attracted tremendous attention due to their unique properties such as high power density, fast charge–discharge process, and long cycling life, which make them promising candidates for next-generation energy storage devices.^1–3^ Generally, supercapacitors can be classified into two categories based on their energy storage mechanisms:^4^ electric double-layer capacitors (EDLCs) that store charges physically on the surface during the charging process, and pseudocapacitors that store energy through reversible faradaic reactions during the charging process.^2^ Nevertheless, the practical applications of supercapacitors are still limited due to their low energy densities.^5,6^ Besides, the rate capabilities and cycle stabilities of pseudocapacitors are usually inferior to those of EDLCs due to insufficient charge transfer and structural instability.^4,7,8^ Therefore, enhancing energy densities as well as improving rate capabilities and cycle stabilities of pseudocapacitors simultaneously is a critically important yet challenging task.^9^

The energy storage performance of a supercapacitor depends greatly on the electrochemical activity and the kinetic feature of the electrode, which can be modulated by controlling the composition and morphology of the electrode.^10–12^ Transition metal compounds have been intensively studied for energy storage applications over decades.^13^ Various metal oxides, hydroxides and sulfides have been developed as electrodes for supercapacitors.^14,15^ Among all available pseudocapacitive materials, Co(OH)_2_ has recently attracted great attention due to its high theoretical specific capacitance (about 3460 F g^−1^) and relatively low cost.^16–18^ Furthermore, it has well-defined electrochemical redox activity and unique layered structure with large interlayer spacing, which can facilitate the diffusion of electrolyte into electroactive material.^7^ To date, Co(OH)_2_ and its composites with various morphologies have been synthesized through various methods such as electrodeposition and hydrothermal reactions.^19–22^ Nonetheless, the capacitances of Co(OH)_2_ based electrodes reported in literatures are still far lower than its theoretical value,^23–26^ which is primarily due to the low electronic conductivity and insufficient active sites of the electrode materials that result in sluggish electrode kinetics and low specific capacitances and low rates.^26,27^ A conventional approach to improve the transport of electrons is to involve conductive additives and polymer binders with the active material powders, so as to improve the conductivity of the electrode. But the introduction of non-active materials inevitably compromise the energy density of the electrode.^28^ Another approach is to use conductive substrates (*e.g.* nickel foam, carbon clothes, graphene, and carbon nanotube, *etc*.) as the current collector to fabricate free-standing electrodes, in which the conductive substrates act as a highly porous conductive network, enabling better access of ions and electrons to the active sites, leading to enhanced electrode performances.^25,29,30^ For example, Hercule *et al.* synthesized Co(OH)_2_ sheets on nickel foam substrate with high specific capacitance and excellent cycle stability.^31^ Wang *et al.* grew Co(OH)_2_ needle arrays on carbon nanotube foams and the composite exhibited higher specific capacitance and improved rate capability.^24^ However, reticular conductive backbones such as nickel foam and stainless steel mesh are not satisfactory due to their limited specific surface area which results in depreciated volumetric energy density of the electrode.^32^ Nanostructured carbon based scaffolds still face the challenge of distributing the inorganic active materials evenly on the substrates in a facile method due to the weak interaction between the inorganic active materials and the carbon scaffold.^33^ Therefore, it is imperative to develop a technology that is able to control the size and morphology as well as the distribution of Co(OH)_2_ on the substrate to make full utilization of interstitial spaces and active materials. With all these considerations, we propose the controlled growth of thin Co(OH)_2_ nanoflake arrays on a well-aligned ultrafine reticular metallic conductive substrate to form a hierarchical electrode through a hydrothermal method. This structure can not only mitigate the contact resistance of the substrate and the active materials and facilitate the transport of electrons and shorten the transport distance of ions, but also improve the electrode–electrolyte contact, which is of great assistance to improve the performance of the electrode.^34–37^ Nonetheless, it is a challenge to achieve such an electrode with a high mass loading of active materials.

Here in this work, by using a facile one-step surfactant-free hydrothermal method, we successfully grew thin Co(OH)_2_ nanoflakes with open porous structure on 1D well-ordered, highly-conductive and hydrophilic NNA substrate,^38^ and obtained the NNACOH composite electrode with metallic hierarchical array structure. The lavender-structured composite electrode showed both a high mass loading and a high specific surface area, and delivered a greatly enhanced specific capacitance with an improved rate capability and excellent cycle stability. An asymmetric supercapacitor was assembled by employing the composite electrode directly as the positive electrode and an activated carbon (AC) electrode as the negative electrode, which not only had excellent mechanical flexibility, but also delivered a high energy density together with a high power density. The superior performance of the electrode could be attributed to the rational design of its structure: by controlling the morphology and porosity of the Co(OH)_2_ nanoflakes grown on NNA, we fabricated the well-ordered, hierarchical, lavender-structured NNACOH electrode with abundant electrochemical active sites, improved electrolyte–electrode contact, and facilitated electron/ion transport, which greatly enhanced the performance of the electrode.

## 2 Results and discussion

**Fig. 1:**
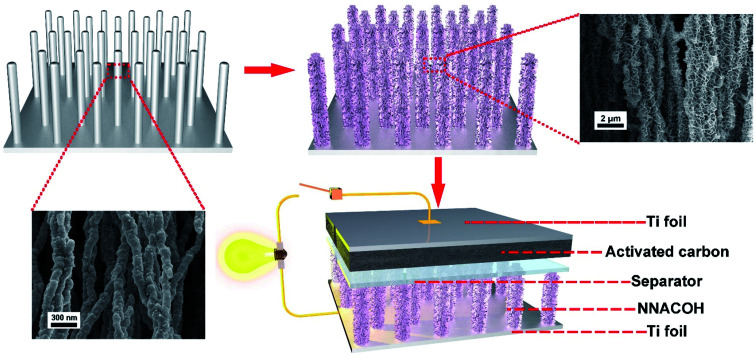
Schematic illustration of the process flow of fabrication of the NNACOH//AC ASC this work. First, Co(OH)_2_ is grown on NNA through hydrothermal reaction. Then the ASC is assembled by using the NNACOH electrode as the positive electrode and the AC electrode as the negative electrode.

Fig. 1 shows the process flow of the fabrication process of the NNA//AC ASC device in this work. The preparation of NNA follows our previous study with a modified condition.^35^ The NNACOH composite electrode was prepared by growing Co(OH)_2_ nanoflakes on NNA through a facile hydrothermal reaction. The mass loading of the active material was measured by calculating the mass change of the NNA before and after the hydrothermal reaction and was measured to be 5.42 mg cm^−2^. An ASC was assembled by employing the NNACOH composite directly as the positive electrode and activated carbon (AC) as the negative electrode (see Experimental section for details).

**Fig. 2:**
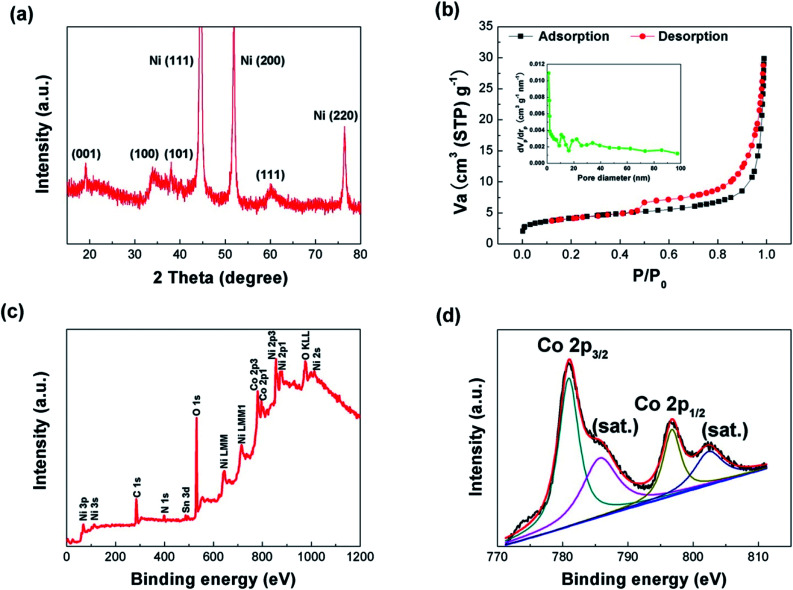
(a) XRD pattern of the NNACOH sample; (b) Nitrogen adsorption–desorption isotherm of the NNACOH sample, the inset showing the pore-size distribution. (c) Full XPS of the NNACOH sample; (d) Co 2p XPS of the NNACOH sample.

X-ray diffraction (XRD) was conducted to investigate the crystallinity of the composite material. Fig. 2a provides the XRD pattern of the NNACOH composite material electrode. The diffraction peaks of the composite can be well indexed to metallic nickel and β-Co(OH)_2_ (JCPDS card no. 30-0443), with the peaks at 44.5°, 51.8° and 76.4° corresponding to the (111), (200) and (220) planes of metallic nickel, and the peaks at 19.1°, 32.5°, 37.9° and 61.5° corresponding to the (001), (100), (101) and (111) planes of β-Co(OH)_2_, which indicates the successful fabrication of the NNACOH composite material electrode. The Brunauer–Emmett–Teller (BET) measurement was conducted to study the porosity of NNACOH and nickel foam@Co(OH)_2_ (NFCOH). Fig. 2b shows the nitrogen adsorption–desorption isotherm of NNACOH. The type-II sorption behaviour with a hysteresis loop in the *P*/*P*_0_ range of 0.4–1.0 indicates that it has multi-model and hierarchical porosity, *i.e.*, with mesopores together with macropores.^24^ The NNACOH sample has a pore volume of 0.46 cm^3^ g^−1^ and a high specific surface area of 74.5 m^2^ g^−1^, which is much higher than NFCOH (specific surface area about 5.8 m^2^ g^−1^ and total pore volume about 0.06 cm^3^ g^−1^, Fig. S1†). X-ray photoelectron spectroscopy (XPS) was conducted to investigate the chemical states of the Co(OH)_2_ electrode material. Fig. 2c shows the full XPS spectrum of NNACOH, from which we can deduce the existence of Ni, Co, and O elements in the composite electrode. Fig. 2d shows the XPS spectrum of Co 2p. The peaks at around 781.5 eV and 797.6 are attributed to the Co 2p_3/2_ and the Co 2p_1/2_, respectively. The XPS spectrum of O 1s of the sample is presented in Fig. S2,† and the peak located at around 532 eV can be assigned to OH^−^. The results in Fig. 2 suggest that β-Co(OH)_2_ is successfully grown on NNA with good crystallinity and controlled porous structure.

**Fig. 3:**
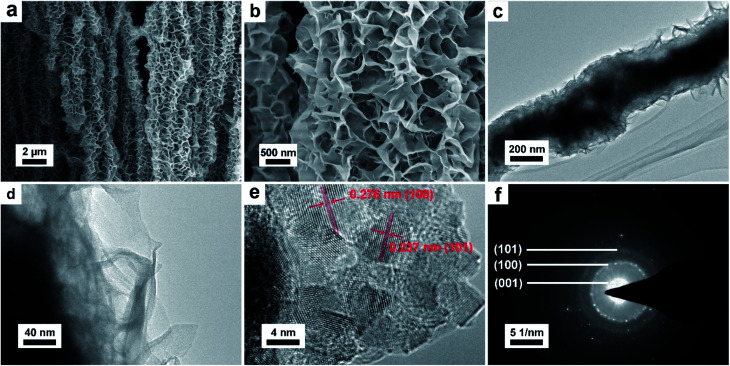
(a) SEM image of the NNACOH sample at low magnification; (b) SEM image of the NNACOH sample at high magnification; (c) TEM image of the NNACOH sample at low magnification; (d) TEM image of the NNACOH sample at high magnification; (e) HRTEM image of the NNACOH sample; (f) SAED pattern of the NNACOH sample.

Scanning electron microscope (SEM) and transmission electron microscope (TEM) were conducted to investigate the morphology and structure of the as prepared NNACOH sample. The SEM image of pristine NNA (Fig. S3†) shows that the surface of NNA is smooth. Fig. 3a shows that the electrode maintains the array-like structure of NNA. Fig. 3b presents the enlarged view of Co(OH)_2_ anchored on NNA, showing that the Co(OH)_2_ are interconnected petal-like thin flakes with abundant pores. Nickel foam was also used as the substrate to grow Co(OH)_2_ through the same method under the same condition (see experimental section). The mass loading of NFCOH is about 5.23 mg cm^−2^, and its SEM image is presented in Fig. S4† for comparison with NNACOH, from which we can see that the Co(OH)_2_ flakes are less uniformly grown on nickel foam, which may result in the loss of activity for the Co(OH)_2_ sheets that do not have good contacts with the substrate. This can be attributed to the lower specific surface area of nickel foam, which results in the planar and smooth surface of nickel foam at nanoscale. This did not happen to NNA, as the surface of NNA is curved at nanoscale, which facilitates the radial growth of Co(OH)_2_ uniformly on NNA. Fig. 3c and d present the TEM images of the NNACOH composite electrode, showing that the composite has a hierarchical structure. The dark area in the middle of the composite is attributed to NNA, and the light area on the fringe of the composite is attributed to Co(OH)_2_ flakes. Fig. 3e presents the HRTEM image of the Co(OH)_2_. The lattice fringes of the (100) plane (0.276 nm) and (101) plane (0.237 nm) can be clearly observed, indicating good crystallinity of the sample. Fig. 3f shows the selected area electron diffraction (SAED) pattern of the Co(OH)_2_. The diffraction rings can be indexed to the (001), (100) and (101) plane of β-Co(OH)_2_ (JCPDS card no. 30-0443), which is in good accordance with the XRD result. The SEM and TEM results reveal the hierarchical structure of the NNACOH composite, with the Co(OH)_2_ flakes covering uniformly on NNA.

**Fig. 4:**
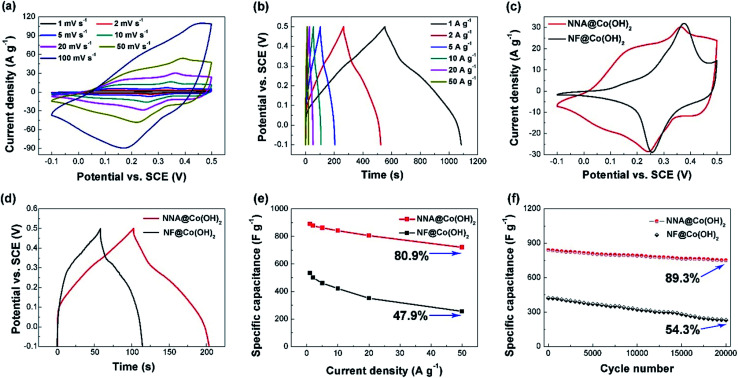
(a) CV curves of the NNACOH electrode at different scan rates; (b) GCD curves of the NNACOH electrode at different current densities; (c) CV curves of the NNACOH electrode and the NFCOH electrode at the scan rate of 20 mV s^−1^; (d) GCD curves of the NNACOH electrode and the NFCOH electrode at the current density of 5 A g^−1^; (e) rate capability of the NNACOH electrode and the NFCOH electrode; (f) cycle stability at the current density of 10 A g^−1^ of the NNACOH electrode and the NFCOH electrode.

The electrochemical performance of the as-synthesized NNACOH composite electrode was evaluated in a three-electrode system, with NNACOH directly used as the working electrode, a platinum foil as the counter electrode, a saturated calomel electrode (SCE) as the reference electrode, and a 1 M KOH aqueous solution as the electrolyte, respectively. The CV curves of the NNACOH electrode with the potential window from −0.10 to 0.50 V at the scan rate ranging from 1 to 100 mV s^−1^ is presented in Fig. 4a. As can be seen, all curves show two pairs of well-defined redox peaks instead of rectangular shape, indicating the pseudocapacitance of the electrode. The CV curves show no distortion even at a high scan rate of 100 mV s^−1^, indicating that the electrode is favorable for fast faradaic reactions. The NNA contributes to negligible capacitance to the NNACOH electrode, which can be seen from the cyclic voltammetry (CV) curves of both the NNACOH electrode and NNA (Fig. S5†). Fig. 4b presents the galvanostatic charge/discharge (GCD) curves of the electrode with the potential window from −0.10 to 0.50 V at the current density ranging from 1 to 50 A g^−1^. The quasi-triangular shape with a plateau at about 0.1 to 0.3 V confirms the pseudocapacitance of the electrode. The plateau can be attributed to the following reversible redox reactions:^39^

1 Co(OH)_2_ + OH^−^ ↔ CoOOH + H_2_O + e^−^

2 CoOOH + OH^−^ ↔ CoO_2_ + H_2_O + e^−^

Fig. 4c presents the CV curves of NNACOH and NFCOH at the scan rate of 20 mV s^−1^. It can be seen that the curve of NNACOH shows a larger enclosed area than that of NFCOH, indicating that NNACOH has higher specific capacitance. The CV curve of NNACOH shows two well-defined pairs of peaks. The first pair of peak at about 0.1 V is attributed to the reversible transition of Co^2+^/Co^3+^ based on reaction (1), and the second pair of peaks at about 0.4 V is attributed to the reversible transition of Co^3+^/Co^4+^ based on reaction (2).^40^ The CV curves of NFCOH showed only one pair of well-defined peaks, with the pair of peaks at about 0.1 V shrinking, which may be due to the formation of Ni/Co hydroxides. With increasing nickel percentage in cobalt hydroxide, the redox peak at about 0.1 V tend to disappear, and the redox peak at about 0.4 V tend to move to higher potential positions.^9^ This does not happen to NNACOH, because the mass of NNA is only 3.13 mg cm^−2^, which is much lower than the mass of NF (18.17 mg cm^−2^). Fig. 4d shows the GCD curves of NNACOH and NFCOH, and the longer discharge time of the NNACOH electrode confirms that it has higher specific capacitance. Fig. 4e shows the capacitances of the electrodes as a function of current density. The capacitance of the NNACOH electrode was calculated to be 891.2, 877.1, 862.5, 841.9, 806.2 and 721.7 F g^−1^ at the current density of 1, 2, 5, 10, 20, 50 A g^−1^, with a capacitance retention of up to 80.9% from 1 to 50 A g^−1^, while the capacitance of the NFCOH electrode was calculated to be only 534.3, 501.5, 460.3, 422.8, 351.6, and 256.1 F g^−1^, respectively, with a capacitance retention of only 47.9% from 1 to 50 A g^−1^ (based on the mass of active materials). The cycle stabilities of the electrodes were measured by repeatedly charging/discharging the electrodes at the current density of 10 A g^−1^ and the results are presented in Fig. 4f. The NFCOH electrode exhibited a relatively poor cycle stability, with a capacitance retention of only 54.3% after 20 000 cycles, while the NNACOH electrode has a high capacitance retention of 89.3%, which is the among the highest for Co(OH)_2_ based electrodes and even comparable to that of EDLCs (see Table S1†). The excellent electrochemical performance of the NNACOH electrode can be ascribed to its unique stable hierarchical open porous structure. NNA act as the robust scaffold to support Co(OH)_2_ flakes, so as to prevent the aggregation of Co(OH)_2_ flakes, and to provide fast electron channels; while thin Co(OH)_2_ flakes with large interlayer spaces act as the active materials which can provide rich redox sites and rapid electrolyte accessibility, shorten the ion diffusion paths, and release strain during faradaic process. All these features endow the composite electrode with fast and high capacity energy storage.^41^

**Fig. 5:**
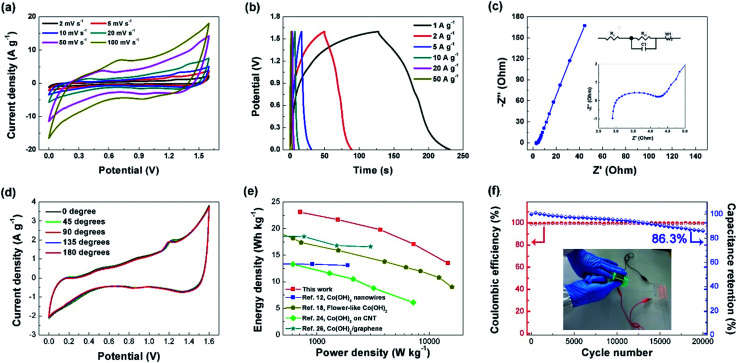
(a) CV plot of the NNACOH//AC ASC at different scan rates; (b) GCD curves of the NNACOH//AC ASC at different scan rates; (c) Nyquist plot of the NNACOH//AC ASC, the insets showing the equivalent circuit and the Nyquist plot in high-frequency region, respectively; (d) CV curves of the NNACOH//AC ASC at the scan rate of 5 mV s^−1^ at different bending angles; (e) Ragone plot of the NNACOH//AC ASC (the energy densities, and power densities are based on the total mass of electrodes); (f) Cycle stability and coulombic efficiency of NNACOH//AC ASC, the inset showing the device powering 12 LEDs in parallel when being bended.

Based on our literature survey (Table S1†), only a few works have reported the performance of the Co(OH)_2_ based supercapacitor device. Here, to evaluate the performance of the composite material at device level, an ASC was assembled with a thickness of about 400 μm by employing the NNACOH electrode directly as the positive electrode, AC electrode as the negative electrode, 1 M KOH aqueous solution as the electrolyte, and glass fiber as the separator, respectively. The working potential of the device can be extended to 1.6 V. The device was tested in a two-electrode system. The CV plot of the AC electrode is presented in Fig. S7.† The quasi-rectangular shape indicates EDLC behaviour of the electrode. The capacitance of the AC electrode is calculated to be 74 F g^−1^ at 1 mV s^−1^. Fig. 5a shows the representative CV plot of the ASC with the potential window from 0 to 1.6 V at scan rates ranging from 2 to 100 mV s^−1^. It can be seen that the ASC exhibits excellent capacitive behavior with contributions from both the NNACOH and the AC electrode. The CV curve showed no distortion even at a high scan rate of 100 mV s^−1^, indicating good rate capability of the device. The GCD curves of the ASC are shown in Fig. 5b. The quasi-triangular shape with good symmetry indicates good reversibility of the device. Electrochemical impedance spectroscopy was conducted to investigate the resistance of the device, and the Nyquist plot of the device is presented in Fig. 5c, with the inset on the top showing the equivalent circuit consisting of a series resistance (*R*_s_), a charge transfer resistance (*R*_ct_), a capacitive element (*C*1), and a Warburg impedance (*W*), and the inset in the middle showing the Nyquist plot of the device in high-frequency region. The simulated *R*_e_ and *R*_ct_ of the device are only 3.1 Ω and 1.4 Ω, respectively, indicating low contact resistance and excellent charge transfer ability of the device. This could be attributed to the fact that the hierarchical structure of the NNACOH electrode provides an appropriate structure for both electronic conduction and ionic transportation.^42,43^Fig. 5d shows the CV curves of the device at different bending angles. The shapes of the CV curves stayed almost unchanged at bending angles from 0–180°, indicating excellent mechanical flexibility of the device, which makes it promising for applications in various fields such as the flexible electronic devices.^44,45^ Furthermore, the device can deliver a maximum energy density of 23.1 W h kg^−1^ at the power density of 712 W kg^−1^ and still an energy density of 13.5 W h kg^−1^ at the maximum power density of 14.7 kW kg^−1^ (based on the total mass of electrodes), which outperforms the flower-like Co(OH)_2_, the Co(OH)_2_ nanowires, Co(OH)_2_ on carbon nanotube (CNT) and Co(OH)_2_/graphene *etc.* ever reported in literatures (see the Ragone plot in Fig. 5e), indicating that the NNACOH electrode is beneficial to achieve better electrode properties and device functions. The cycle stability and coulombic efficiency of the device was tested by repeatedly charging and discharging the device at the current density of 10 A g^−1^ for 20 000 cycles, and the results are shown in Fig. 5f, with the inset showing the device powering twelve light-emitting diodes in parallel. The device has a remarkable cycle stability of about 86.3% after 20 000 cycles, with coulombic efficiencies of nearly 100% in each cycle, which indicates that the device has a long operation life.

To further demonstrate the performance of the ASC for practical applications, two ASCs in parallel and two ASCs in series are connected with each other and tested by GCD. Fig. S8a† shows that when the two ASCs are connected in series under the same working potential of 1.6 V, a doubled charging/discharging duration can be obtained. On the other hand, the working potential can be extended to about 3.2 V (Fig. S8b†) when the two ASCs are connected in series.

The excellent performance of the electrode and the device can be attributed to the rational structural design of the NNACOH electrode structure. By using a facile hydrothermal method to uniformly grow Co(OH)_2_ nanoflakes on the top of NNA scaffold, and to control the morphology and porosity of the electrode structure, a lavender-like hierarchical electrode structure is obtained with a high mass loading and large specific surface area. The electrode can not only provide abundant electrochemical active sites and mitigate the contact resistance between the substrate and the active materials, but also facilitate electron/ion transport and releases stress during redox reactions, which can critically enhance its performance.

## 3 Experimental section

All chemicals are acquired from Alfa Aesar and directly used without further purification. The detailed synthesis process is as follows.

### 3.1 Synthesis of NNA

The NNA is synthesized *via* a template-free method. Typically, a piece of Ti foil was deposited with an active seed layer of Pd through electroless plating. Ni nanoparticles were generated through the reaction of NiCl_2_ solution and hydrazine at the temperature of 80 °C, and the Ni nanoparticles were assembled onto the Pd layer by applying a magnetic field vertical to the Ti foil. After reacting for 60 min, nickel nanowire arrays were successfully grown vertically on Ti foil.

### 3.2 Fabrication of NNACOH

The NNACOH electrode sample was synthesized through hydrothermal method. In a typical procedure, 1.5 mmol Co(NO_3_)_2_·6H_2_O and 2.2 mmol hexamethylenetetramine (HMTL) were dissolved into 30 mL deionized water by continuous stirring to form a transparent pink solution. The solution was transferred to a 50 mL autoclave and, followed by immersing a piece of NNA (2 cm × 2 cm) into the solution. The autoclave was then sealed and heated at 120 °C for 12 h. Then the sample was taken out and washed with deionized water and ethanol for several times and dried at 50 °C in air overnight.

### 3.3 Fabrication of NFCOH

The NNACOH was synthesized using a method similar to the fabrication of NNACOH. NNA was replaced by a piece of nickel foam (2 cm × 2 cm) in the hydrothermal reaction.

### 3.4 Fabrication of the activated carbon electrode

The AC electrode was prepared by mixing commercial AC, acetylene black and PVDF with a mass ratio of 7 : 2 : 1 in NMP and then dispensed on Ti foil.

### 3.5 Fabrication of NNACOH//AC ASC

The NNACOH//AC ASC was assembled by using NNACOH as the positive electrode and AC electrode as the negative electrode.

The masses of the positive and negative electrodes were balanced according to the equation below:3
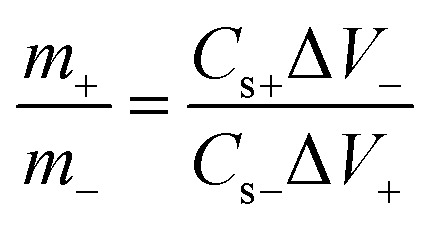
where *m*_+_ and *m*_−_ are the masses of the positive electrode and the negative electrode, respectively; *C*_s+_ and *C*_s−_ are the specific capacitances of the positive electrode and the negative electrode, respectively; Δ*V*_+_ and Δ*V*_−_ are the voltage ranges of the positive electrode and the negative electrode, respectively.

### 3.6 Material characterizations

The crystal structure of the samples was characterized by X-ray powder diffraction patterns (XRD, Rint-2000V/PC, Rigaku, Japan). The morphology of the sample was studied by Field-Emission Scanning Electron Microscopy (FE-SEM, Carl Zeiss, ZEISS SUPRA55). The structure of the sample was investigated Field-Emission Transmission Electron Microscopy (FEI Tecnai G^2^ F30). X-ray photoelectron spectroscopy (XPS) was conducted on X-ray photon-electron spectrometer (XPS, Thermo Fisher, ESCALAB 250X). The textural characterizations of the samples were performed by nitrogen adsorption at 77 K in a micromeritics ASAP 2020 apparatus, and the specific surface areas were measured *via* Brunauer–Emmett–Teller (BET) method.

### 3.7 Electrochemical measurements

The electrochemical properties of the single electrodes were measured at room temperature in three-electrode configuration in 1 M KOH solution, with NNACOH as the working electrode, a Pt foil and a saturated calomel electrode as the counter electrode and the reference electrode, respectively. The electrochemical measurements of devices were conducted in two-electrode configuration with NNACOH as the positive electrode and AC as the negative electrode. The electrochemical properties of the samples were all evaluated using a VMP3 electrochemical working station.

The capacitances of the active materials were calculated from GCD curves according to the following equations:4
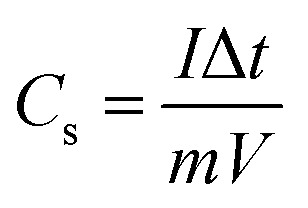
where *I* is the current density, Δ*t* is the discharge time, *m* is the mass of active materials, *V* is the potential range, and *C*_s_ is the specific capacitance of the electrode.

The capacitances of the devices were calculated from GCD curves according to the following equations:5
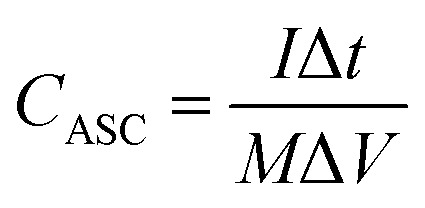
where *I* is the current density, Δ*t* is the discharge time, *M* is the total mass of positive and negative materials (*M* = *m*_+_ + *m*_−_), *V* is the potential range, and *C*_ASC_ is the specific capacitance of the asymmetric supercapacitor.

The calculations of energy and power density of ASC are based on the total weight of the two electrodes in the ASC according to the following equations:6
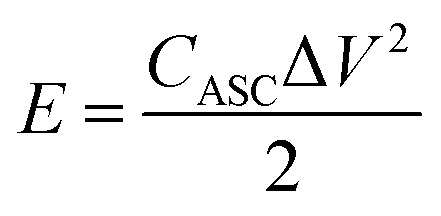
7
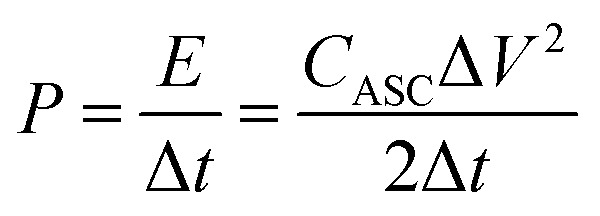
where *E* is the energy density, *P* is the power density, *C*_ASC_ is the capacitance of the asymmetric supercapacitor, Δ*V* is the potential range, and Δ*t* is the discharge time.

Electrochemical impedance spectroscopy (EIS) measurements were made in the frequency range of 0.01–100 000 Hz by applying an alternating current voltage with 5 mV perturbation.

## 4 Conclusion and outlook

In summary, we synthesized the NNA@Co(OH)_2_ composite electrode by growing Co(OH)_2_ nanoflakes on highly conductive NNA through a facile hydrothermal method, and obtained the NNACOH composite with high specific surface area. The composite electrode exhibited a high specific capacitance of 891.2 F g^−1^ and a high rate capability with a capacitance retention of 80.9% from 1 to 50 A g^−1^. The electrode also exhibited an excellent cycle stability, with a capacitance retention of 89.4% after 20 000 cycles at 10 A g^−1^. An asymmetric supercapacitor consisting of the NNACOH cathode coupled with an AC anode delivered a high energy density of 23.1 W h kg^−1^ at the power density of 712 W kg^−1^ and still an energy density of 13.5 W h kg^−1^ at the maximum power density of 14.7 kW kg^−1^ (based on the total mass of the electrodes). In addition, it showed excellent mechanical flexibility, which made it promising for practical applications in various energy storage fields. The excellent performance of the electrode could be ascribed to its stable and open-porous hierarchical structure, which not only facilitated the transport of electrons and ions, but also contributed to the release of strain during charging/discharging process. In short, by rationally designing the structure and elaborately controlling the fabrication process of the electrode, we successfully boosted the energy storage performance characteristics significantly for the Co(OH)_2_ based electrode system. We expect that our work will also inspire development of other pseudocapacitive electrode structures for energy storage applications.

## Conflicts of interest

There are no conflicts to declare.

## Supplementary Material

RA-008-C8RA02844C-s001
