# Supplementary material for: Lavender-like cobalt hydroxide nanoflakes deposited on nickel nanowire arrays for high-performance supercapacitors
Source: RSC Adv. 2018 May 11;8(31):17263–71. doi: 10.1039/c8ra02844c (PMC9080414; doi:10.1039/c8ra02844c)
Supplement: RA-008-C8RA02844C-s001 [file RA-008-C8RA02844C-s001.pdf]

Electronic Supplementary Material (ESI) for RSC Advances.
This journal is © The Royal Society of Chemistry 2018

Supporting information

Lavender-like cobalt hydroxide nanoflakes deposited on nickel nanowire arrays for high-performance supercapacitors

Jie Liao,[a] Xuanyu Wang,[a] Yang Wang,[a] Songyang Su,[a] Adeela Nairan,[a] Feiyu Kang,[a, b] and Cheng Yang*[a]

[a]Division of Energy and Environment, Graduate School at Shenzhen, Tsinghua University, Shenzhen, 518055, China.

[b]School of Materials Science and Engineering, Tsinghua University, Beijing 100084, China.

Corresponding Author: yang.cheng@sz.tsinghua.edu.cn

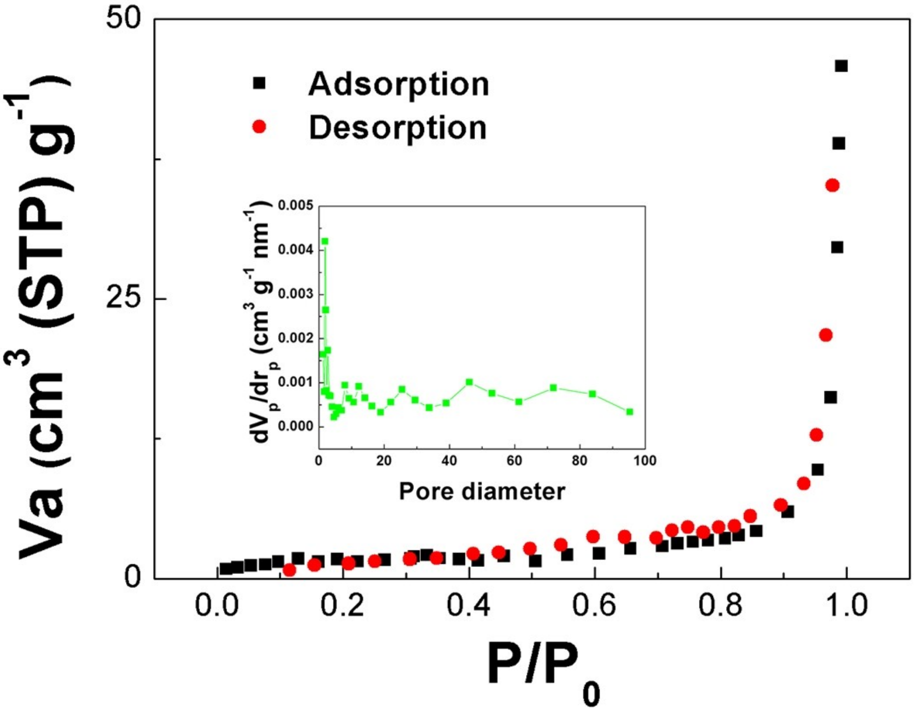

Figure S1. Nitrogen adsorption-desorption isotherm of the NFCOH sample, the inset showing the pore-size distribution.

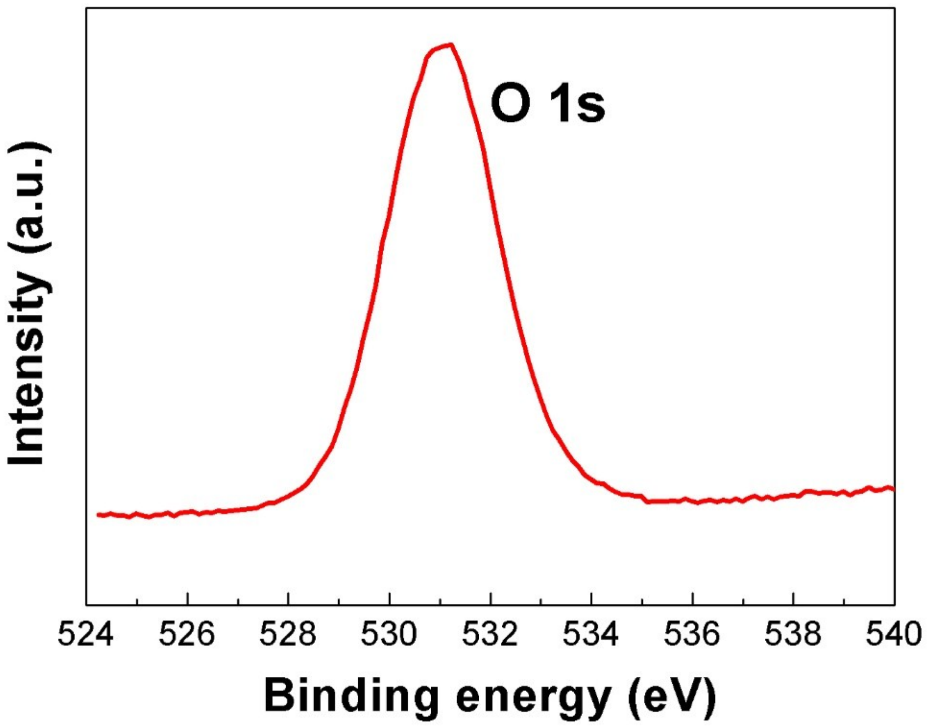

Figure S2. X-ray photon spectrum of O1s of the NNA@$Co(OH)_2$ sample.

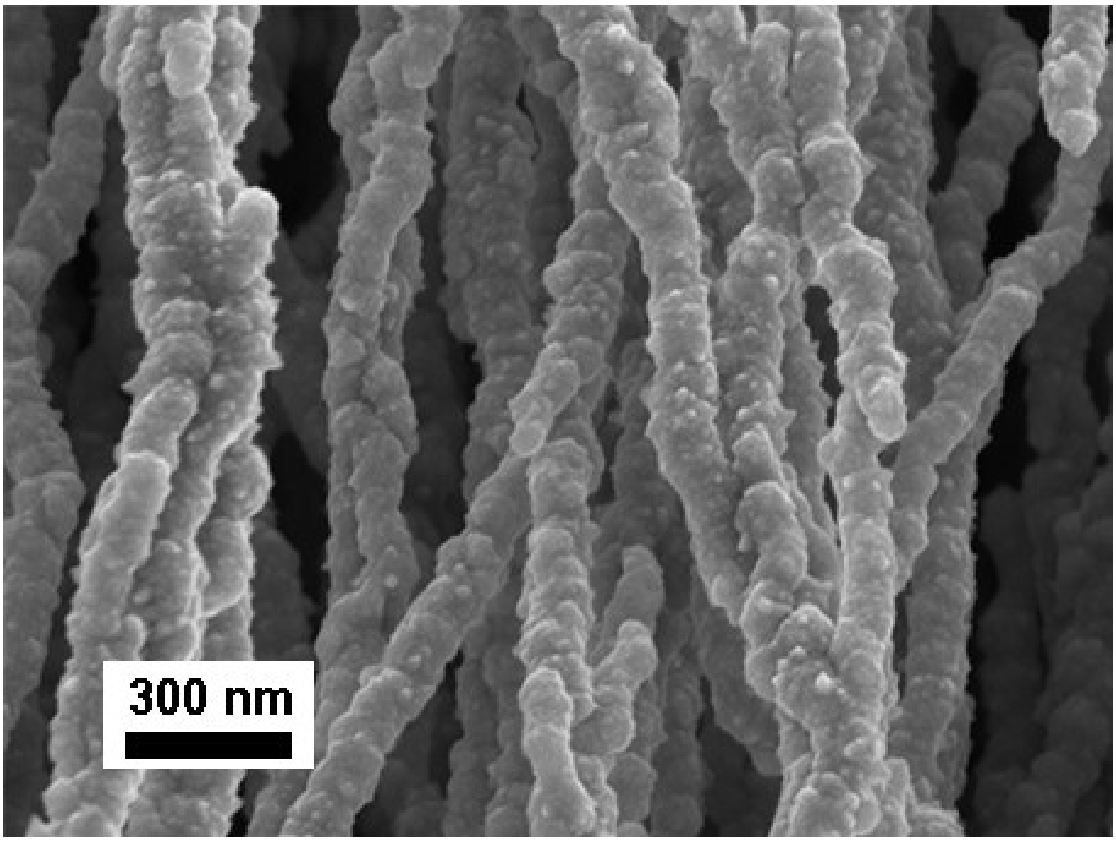

Figure S3. SEM image of the pristine nickel nanowire arrays.

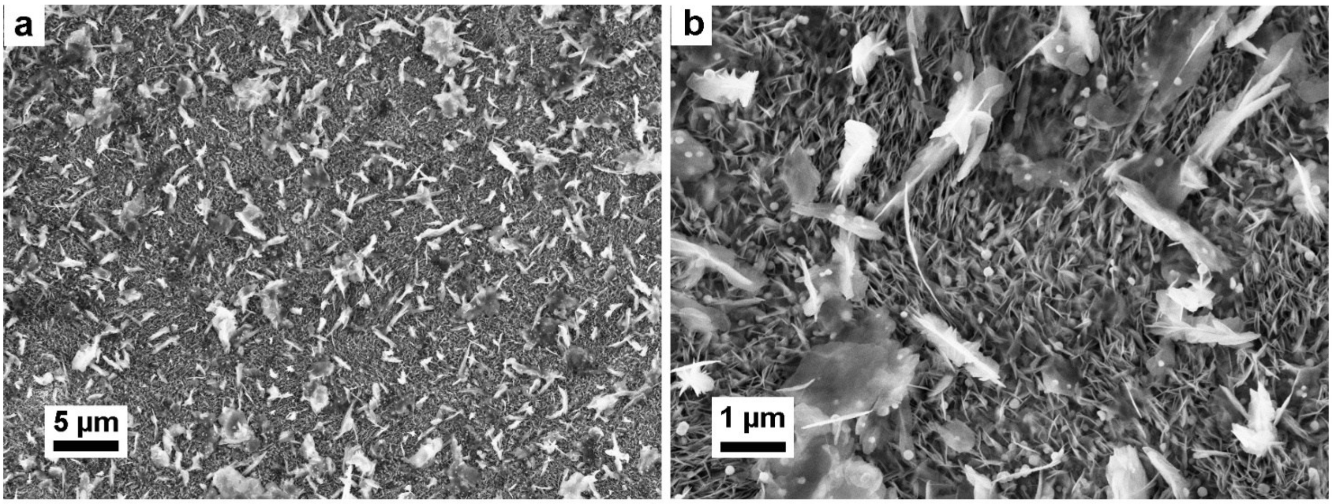

Figure S4. SEM image of nickel foam@$Co(OH)_2$ at different magnifications.

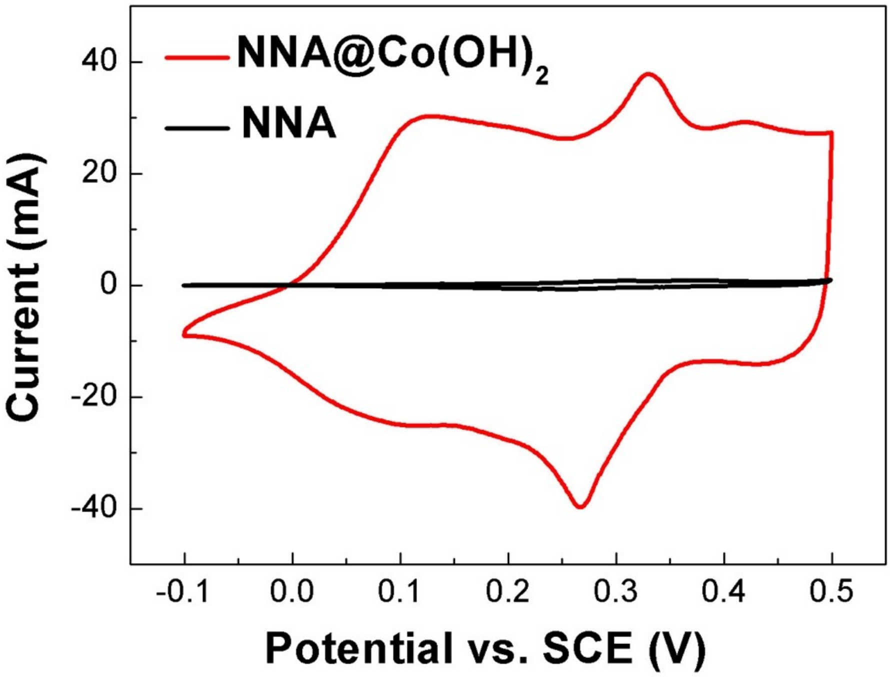

Figure S5. Cyclic voltammetry of the NNA@$Co(OH)_2$ electrode and NNA at the current density of 5 mV s^{-1}.

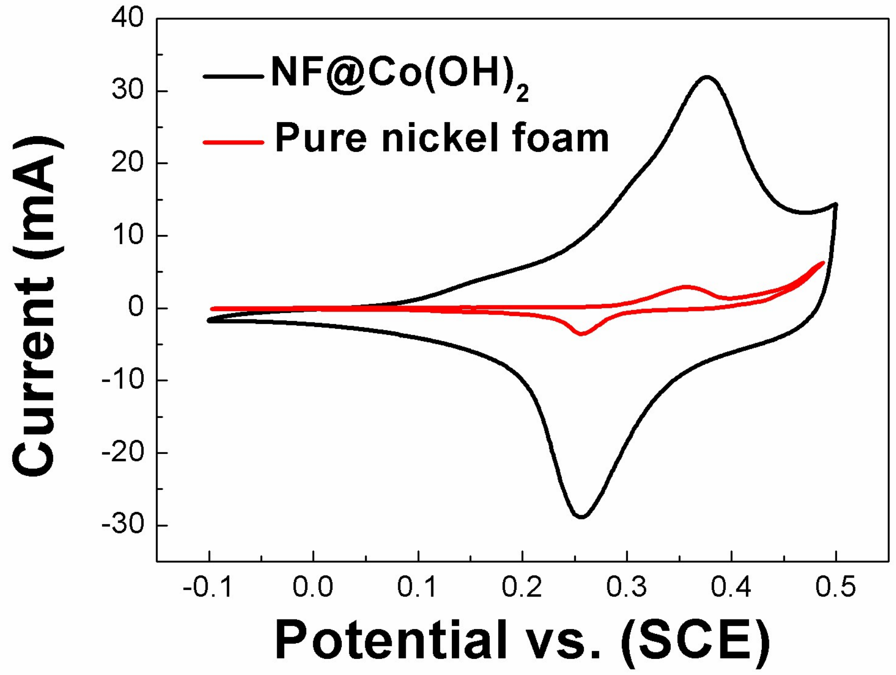

Figure S6. Cyclic voltammetry of the NF@$Co(OH)_2$ electrode and pure nickel foam at the current density of 5 mV s^{-1}.

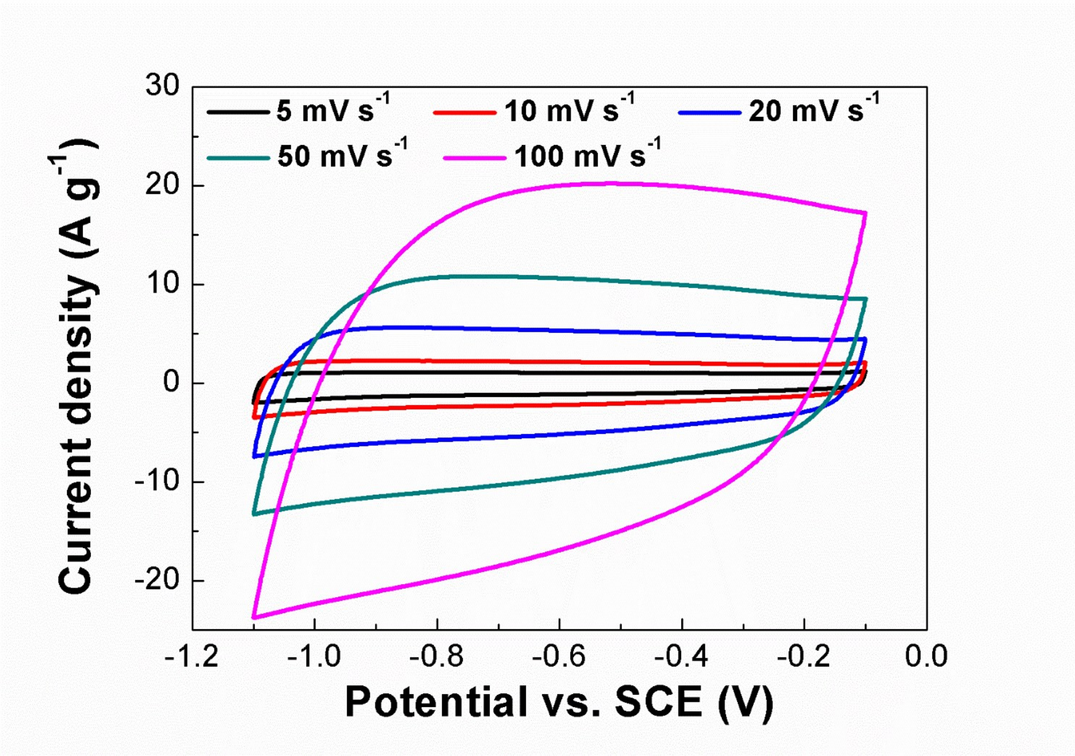

Figure S7. Cyclic voltammetry plot of the activated carbon electrode at the scan rate from 5 to 100 mV s^{-1}.

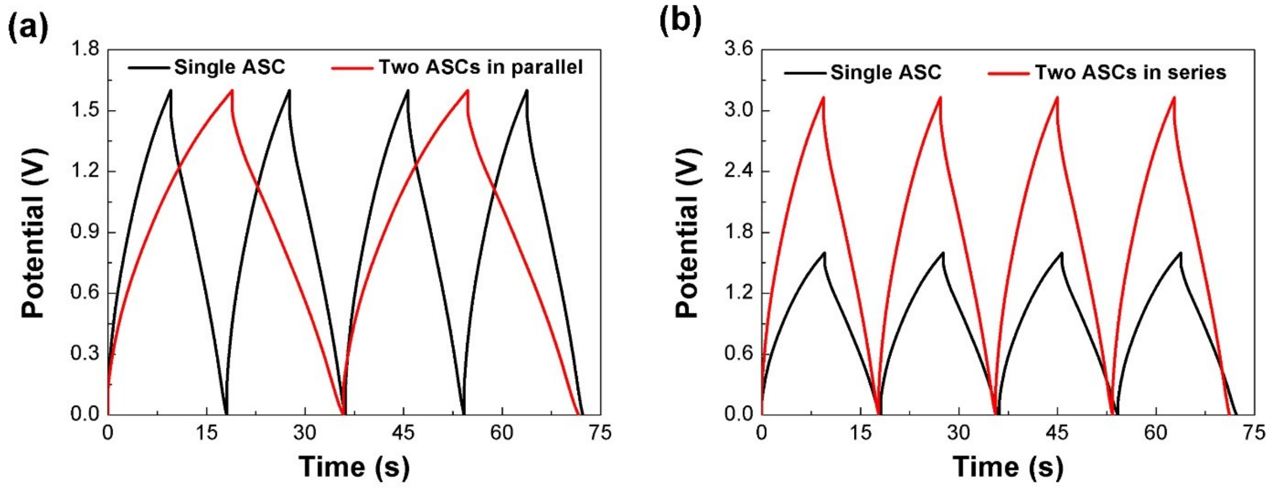

Figure S8. Galvanostatic charge/discharge curves of: a) a single asymmetric supercapacitor (ASC) and two ASCs in parallel, and b) a single ASC and two ASCs in tandem.

Table S1. Electrochemical performances of $Co(OH)_2$ based electrodes from recent reports.

Ref	Morphology	Capacitance at current density	Cycling stability	Energy density at Power density
This work	$Co(OH)_2$ nanosheets on nickel nanowire	891.2 F/g at 1 A/g, 721 F/g at 50 A/g.	89.3% after 20,000 cycles	23.1 Wh kg^{-1} at 712 W kg^{-1}, 13.5 Wh kg^{-1} at 14.7 kW kg^{-1}.
1	$Co(OH)_2$ nanowires	358 F/g at 0.5 A/g, 325 F/g at 10 A/g.	86.3% after 5,000 cycles	13.6 Wh kg^{-1} at 153 W kg^{-1}, 13.1 Wh kg^{-1} at 1.88 kW kg^{-1}.
2	$Co(OH)_2$ arrays on carbon nanotube foam	614 C/g at 0.5 A/g, 425 C/g at 10 A/g.	none	13.3 Wh kg^{-1} at 612 W kg^{-1}, 6.1 Wh kg^{-1} at 7.2 kW kg^{-1}.
3	Flower-like $Co(OH)_2$	429 F/g at 1 A/g, 337 F/g at 10 A/g.	>80% after 4,000 cycles	22 Wh kg^{-1}, 9 Wh kg^{-1} at 15.9 kW kg^{-1}.
4	$Co(OH)_2$/graphene	693.8 F/g at 2 A/g, 506.2 F/g at 32 A/g.	91.9% after 3,000 cycles	19.3 Wh kg^{-1} at 187.5 W kg^{-1}, 16.7 Wh kg^{-1} at 3,000 W kg^{-1}.
5	$Co(OH)_2$ nanosheets	604 F/g at 5 mV/s, 454 F/g at 50 mV/s.	76% after 500 cycles	none
6	$Co(OH)_2$ sheets	885 F/g at 1 A/g, 699 F/g at 10 A/g.	91% after 1,500 cycles	none
7	$Co(OH)_2$ nanocone	562 F/g at 2 A/g, 377 F/g at 32 A/g.	97% after 5,000 cycles	none
8	Co@$Co(OH)_2$ core-shell structure	525 F/g at 0.5 A/g, 396 F/g at 2 A/g.	81.5% after 3,000 cycles	none
9	Graphene/$Co(OH)_2$	474 F/g at 1 A/g, 300 F/g at 10 A/g.	90% after 1,000 cycles	none
10	$Co(OH)_2$ on nickel foam	3.17 F/cm^2 at 5 mA/cm^2.	303% after 2,000 cycles	none
11	$Co(OH)_2$ nanowires	1.44 F/cm^2 at 1 mA/cm^2, 0.99 F/cm^2 at 10 mA/cm^2.	93.6% after 5,000 cycles	none

References:

1. Y. Tang, Y. Liu, S. Yu, S. Mu, S. Xiao, Y. Zhao and F. Gao, *J. Power Sources*, 2014, **256**, 160-169.
2. C. Wang, H. Qu, T. Peng, K. Mei, Y. Qiu, Y. Lu, Y. Luo and B. Yu, *Electrochim. Acta*, 2016, **191**, 133-141.
3. R. Wang, X. Yan, J. Lang, Z. Zheng and P. Zhang, *J. Mater. Chem. A*, 2014, **2**, 12724-12732.
4. C. Zhao, F. Ren, X. Xue, W. Zheng, X. Wang and L. Chang, *J. Electroanal. Chem.*, 2016, **782**, 98-102.
5. T. Zhao, H. Jiang and J. Ma, *J. Power Sources*, 2011, **196**, 860-864.
6. P. Dai, T. Yan, L. Hu, Z. Pang, Z. Bao, M. Wu, G. Li, J. Fang and Z. Peng, *J. Mater. Chem. A*, 2017, **5**, 19203-19209.
7. F. Cao, G. X. Pan, P. S. Tang and H. F. Chen, *J. Power Sources*, 2012, **216**, 395-399.
8. Y. K. Kim, S. I. Cha and S. H. Hong, *J. Mater. Chem. A*, 2013, **1**, 9802.
9. Z. Li, J. Wang, L. Niu, J. Sun, P. Gong, W. Hong, L. Ma and S. Yang, *J. Power Sources*, 2014, **245**, 224-231.
10. Z. Yu, Z. Cheng, X. Wang, S. X. Dou and X. Kong, *J. Mater. Chem. A*, 2017, **5**, 7968-7978.
11. D. Yu, Y. Wang, L. Zhang, Z.-X. Low, X. Zhang, F. Chen, Y. Feng and H. Wang, *Nano Energy*, 2014, **10**, 153-162.
